# An Extended Time-Mode Digital Pixel CMOS Image Sensor for IoT Applications

**DOI:** 10.3390/s25237228

**Published:** 2025-11-26

**Authors:** Taehyoung Kim, Gunhee Han

**Affiliations:** School of Integrated Technology, Yonsei University, Seoul 03722, Republic of Korea; t.kim@yonsei.ac.kr

**Keywords:** CMOS image sensor, digital pixel, low-power CIS, in-pixel comparator, dynamic range, time-mode digitization

## Abstract

Time-mode digital pixel sensors have several advantages in Internet-of-Things applications, which require a compact circuit and low-power operation under poorly illuminated environments. Although the time-mode digitization technique can theoretically achieve a wide dynamic range by overcoming the supply voltage limitation, its practical dynamic range is limited by the maximum clock frequency and device leakage. This study proposes an extended time-mode digitization technique and a low-leakage pixel circuit to accommodate a wide range of light intensities with a small number of digital bits. The prototype sensor was fabricated in a 0.18 μm standard CMOS process, and the measurement results demonstrate its capability to accommodate a 0.03 lx minimum light intensity, providing a dynamic range figure-of-merit of 1.6 and a power figure-of-merit of 37 pJ/frame·pixel.

## 1. Introduction

CMOS image sensor (CIS) technology has advanced significantly over the past two decades, primarily driven by the rapid expansion of mobile-phone applications. Key breakthroughs in this field include the introduction of the four-transistor active pixel sensor (4T-APS) [1], which reduces readout noise, and the back-side illumination (BSI) process [2], which enhances sensitivity, resolution, and dynamic range (DR). As a result, modern CISs now surpass the human eye in terms of sensitivity, resolution, minimum illumination, and frame rate [3,4,5,6,7].

For Internet-of-Things (IoT) applications, CISs must be capable of producing recognizable images of the specific object of interest under poorly illuminated conditions, which requires a wide DR and flexible operation while minimizing complexity and power consumption. Despite the improvements in CIS technology, the DR of CISs remains constrained by the supply voltage. As conventional 4T-APS CISs undergo a conversion chain from light intensity to charge at the photodiode (PD), to voltage at the floating diffusion (FD), and finally to a digital output, each stage must be designed to accommodate the full range of signals. However, the DR is mainly determined by the pinning potential of the PD. Additional bottlenecks arise from analog readout circuits and subsequent analog-to-digital conversion. Photon-counting techniques have been proposed for high sensitivity and binary signal generation to overcome the power-supply limitation [8,9]. However, their practical DR is limited by device uniformity.

As an alternative, time-mode digitization (TMD) has been proposed to represent light intensity as a moment of an event in the time domain rather than as voltages [10,11,12]. This approach eliminates analog circuitry, providing low-power, low-noise, and easy-design features. Since TMD image sensors directly convert light intensity into digitized time, the theoretical DR is determined by the number of bits for the time-measuring counter. However, the practical DR is limited due to the maximum clock frequency and device leakage.

This work proposes an extended TMD (E-TMD) scheme and a low-leakage pixel circuit to accommodate a wide range of illumination intensities with a small digital bit depth. The remainder of this paper is organized as follows: Section 2 introduces the proposed extension of TMD. Section 3 presents the circuit implementation of the proposed digital pixel sensor and simulation results. Section 4 presents the measurement results. Finally, Section 5 concludes the paper.

## 2. Time-Mode Digitization and Its Extension

**Figure 1:**
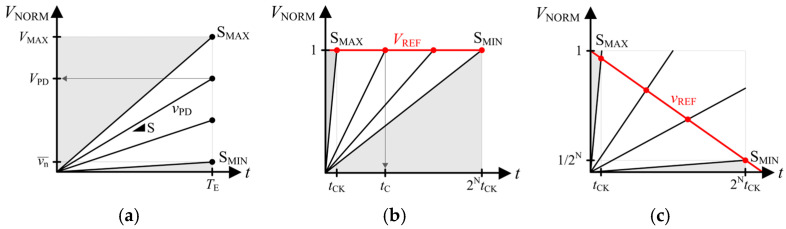
Operation principle of conventional CISs: (**a**) voltage mode; (**b**) fixed reference; and (**c**) ramp-down TMD.

Conventional CISs expose the PD to the incident light for a fixed exposure time *T*_E_. The light-induced PD voltage *v*_PD_ increases linearly with slope S, which is proportional to the light intensity, as shown in Figure 1a. The PD voltage at the end of the exposure time *V*_PD_ is obtained as follows:

(1) $$V_{PD}=ST_{E},\;\frac{\bar{v_{n}}}{T_{E}}\leq S\leq \frac{V_{MAX}}{T_{E}}$$

The voltage-mode digitization (VMD) CIS samples and digitizes the PD voltage at the end of the exposure time. Therefore, the minimum detectable slope is determined by the noise voltage $\bar{v_{n}}$, which includes the circuit noise and the quantization noise. The shaded area in Figure 1 represents the undetectable slope range. Then, the DR is defined as a ratio of S_MAX_ to S_MIN_. The DR of VMD CIS is determined by the analog-to-digital converter (ADC) resolution that accommodates the signal range of [$\bar{v_{n}}$, *V*_MAX_] to N-bit data, providing a DR of 2*^N^*.

TMD digitizes the light intensity by measuring the time until the light-induced PD voltage crosses the reference voltage, as shown in Figure 1b. The resulting crossing time *t*_C_ for a fixed reference voltage *V*_REF_ normalized to 1 is inversely proportional to the light intensity:

(2) $$t_{C}=\frac{1}{S},\;\frac{1}{2^{N}t_{CK}}\leq S\leq \frac{1}{t_{CK}}$$

where *t*_CK_ is the clock period, and N is the number of bits of the time-measuring down counter. High-intensity light whose crossing time is shorter than the time resolution *t*_CK_ cannot be discriminated, whereas low-intensity light that does not cross the reference until the end of counting cannot be detected. Therefore, the DR of the fixed-reference TMD (F-TMD) is bounded by 2*^N^*.

The lower bound S_MIN_ can be extended by ramping down the reference voltage *v*_REF_, as shown in Figure 1c [11]. Then, the crossing time and detectable slope range can be obtained as follows:

(3) $$t_{C}=\frac{2^{N}t_{CK}}{2^{N}t_{CK}S+1},\;\frac{1}{2^{2N}t_{CK}}\leq S≲\frac{1}{t_{CK}}$$

Since the minimum detectable slope is extended to (1/2*^N^*)/(2*^N^t*_CK_), the theoretical DR of ramp-down TMD (R-TMD) is given as 2^2*N*^, which is equivalent to doubling the effective number of bits when compared with the F-TMD or VMD.

**Figure 2:**
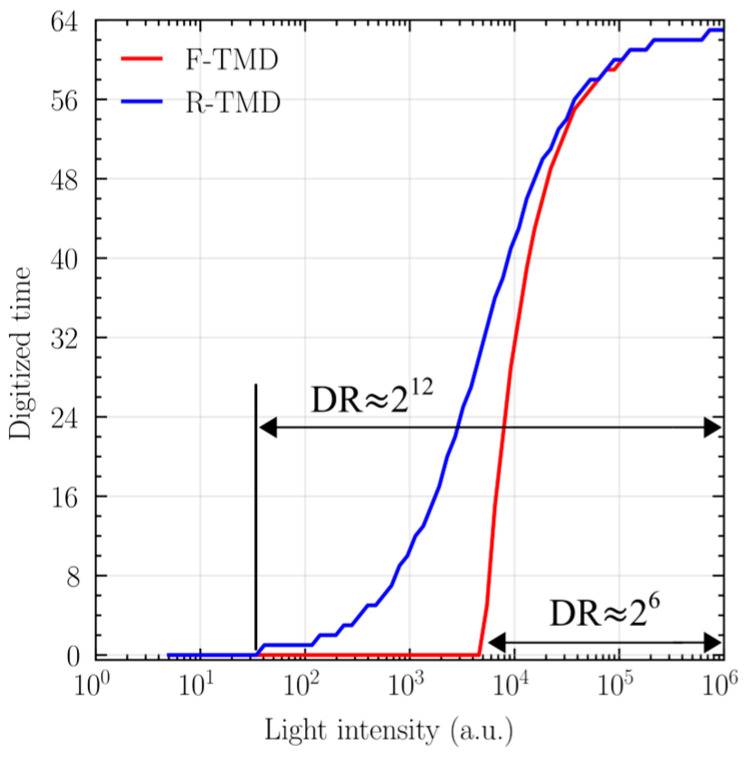
Theoretical photo-transfer curve of conventional 6-bit TMDs.

Although the DR of TMD is determined only by the number of digital bits, it is chosen to be small for compact IoT applications. Figure 2 shows the photo-transfer curves of F-TMD and R-TMD obtained using a 6-bit counter with *t*_CK_ = 0.1 μs. R-TMD extends the detectable lower-intensity bound by mitigating the exaggerated contrast at low illumination. However, contrast saturation in the high-illumination region still remains due to time resolution. The use of high clock frequency can improve the resolution in the high-illumination range, but it simultaneously raises the minimum detectable intensity, leaving the overall DR unchanged.

**Figure 3:**
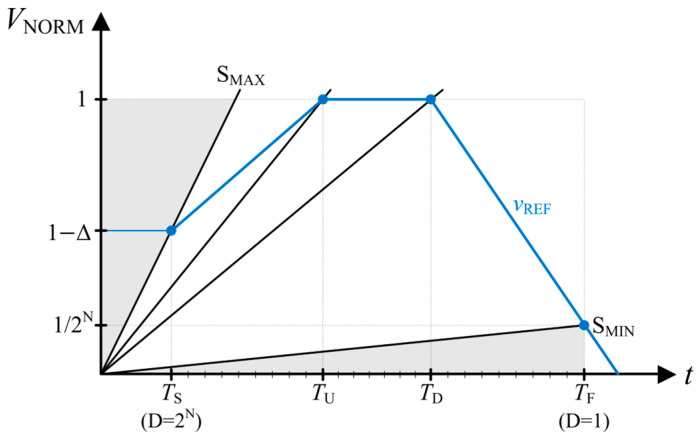
The operation principle of the proposed E-TMD technique.

The dilemma between high contrast at high illumination and minimum detectable intensity can be resolved by introducing a variable clock generator whose frequency decreases as time elapses [12]. Alternatively, the contrast in the high-illumination region can be improved by modifying the reference voltage. The proposed E-TMD technique introduces four phases (no-counting, ramp-up, flat, and ramp-down) with different ramp slopes that are divided by the *T*_S_, *T*_U_, and *T*_D_, as shown in Figure 3. Then, the corresponding crossing time for each phase can be obtained as follows:

(4) $$t_{C}=\left\{\begin{array}{c} \frac{T_{F}}{\left(T_{F}-T_{D}\right)S+1},\;\frac{1}{2^{N}T_{F}}≲S\leq \frac{1}{T_{D}}\\ \;\frac{1}{S},\;\frac{1}{T_{D}}\leq S\leq \frac{1}{T_{U}}\\ \frac{(1-∆{)T}_{U}-T_{S}}{\left(T_{U}-T_{S}\right)S+∆},\;\frac{1}{T_{U}}\leq S\leq \frac{1-∆}{T_{S}}\; \end{array}\right.$$

The counter starts at *T*_S_ and reaches its maximum value 2*^N^* at *T*_F_, dividing the time duration between *T*_S_ and *T*_F_ into 2^N^ sections with intervals of *t*_CK_. The corresponding slope range is then mapped to an N-bit digital value D. The DR of the proposed E-TMD technique can be obtained to be 2^N^*T*_F_/*T*_S_ when Δ = 0. Since IoT image sensors prioritize object recognition over natural intensity rendering, the photo-transfer curve can be tailored using *T*_U_ and *T*_D_ to maximize the contrast for the target object.

**Figure 4:**
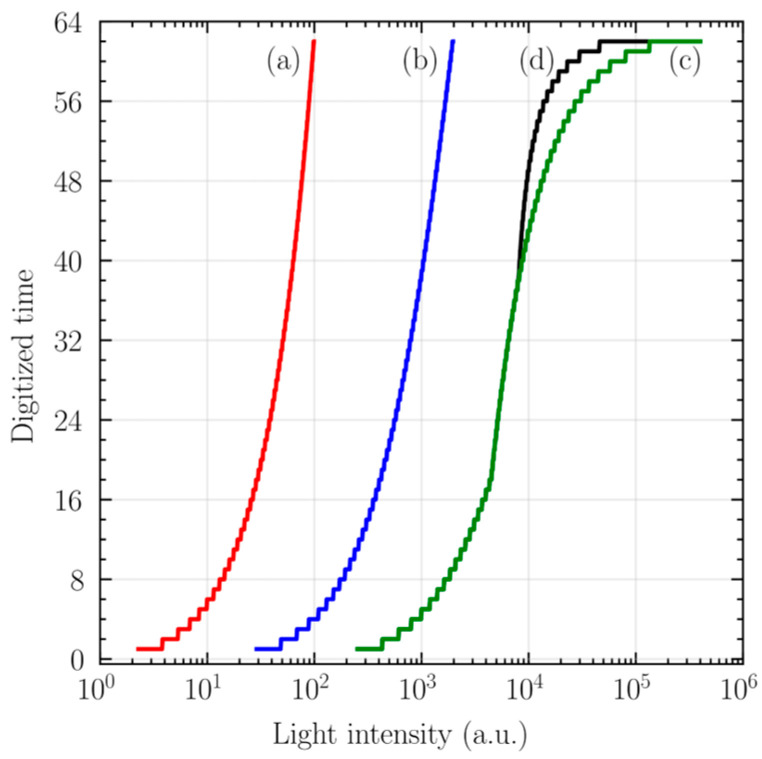
Theoretical photo-transfer curve of the proposed E-TMD: (**a**) *T*_S_ = *T*_U_ = *T*_D_ = 2000*t*_CK_, Δ = 0; (**b**) *T*_S_ = *T*_U_ = *T*_D_ = 100*t*_CK_, Δ = 0; (**c**) *T*_S_ = *T*_U_ = 0, *T*_D_ = 45*t*_CK_, Δ = 0; and (**d**) *T*_S_ = 0, *T*_U_ = 25*t*_CK_, *T*_D_ = 45*t*_CK_, Δ = 0.7.

Figure 4 shows the photo-transfer curves for various choices of *T*_S_, *T*_U_, *T*_D_, *T*_F_, and Δ, obtained using a 6-bit counter with *t*_CK_ = 0.1 μs. Curves (a), (b), and (c) indicate that the maximum endpoint of the transfer curves shifts toward lower illumination as *T*_S_ increases. Note that the contrast in the high-illumination region becomes compressed as *T*_S_ becomes smaller due to the limited time resolution. The high-illumination contrast can be improved by introducing a ramp-up phase, as shown in curve (d), without increasing the clock frequency.

## 3. Low-Leakage Time-Mode Digital Pixel Circuit

**Figure 5:**
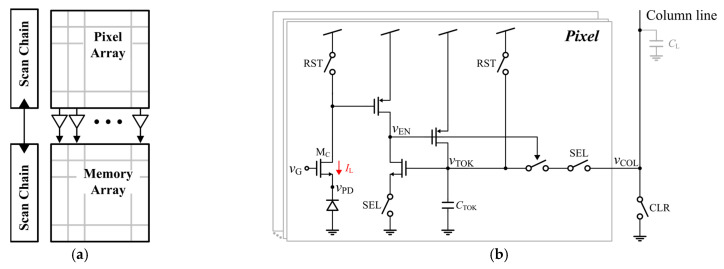
The conventional time-mode digital pixel CIS adapted from [11]: (**a**) architecture; (**b**) pixel schematic diagram.

The TMD pixel sensor [11] consists of a digital pixel array and a pixel-by-pixel mapped embedded frame memory array that latches the counter value according to the binary signal from the corresponding pixel, as shown in Figure 5a. The digital pixel sets the in-pixel token memory when the PD voltage crosses the reference voltage. The token memory status is repeatedly scanned, while the counter value increases at every scan cycle. The counter value is latched into the corresponding memory cell when the token is set, and then the token is reset after the readout so that latching does not occur again.

The previous digital pixel circuit, shown in Figure 5b, uses a dynamic comparator, in which the reference voltage *v*_REF_ is applied by controlling the gate voltage *v*_G_ of the comparator transistor M_C_. The PD voltage *v*_PD_ is reset to *V*_DD_-*V*_T_, where *V*_T_ is the threshold voltage of M_C_, by applying *v*_G_ to *V*_DD_. M_C_ is turned off by lowering *v*_G_ to *V*_DD_-*v*_REF_ during the exposure. M_C_ turns on again when the light-induced PD voltage change exceeds *v*_REF_. Once M_C_ is turned on, *v*_EN_ is kept high, allowing for the token memory to be accessed through the column line. The charge in the token memory is discharged to a pre-cleared column line capacitance *C*_L_, inducing a column line voltage variation when the pixel is selected. This charge sharing occurs only once after the crossing during one frame period. The token capacitor *C*_TOK_ should be sufficiently large (e.g., 5.5 fF) to induce a column line voltage variation that can be detected by the column sense amplifier.

The previous digital pixel suffers from the leakage current *I*_L_ that flows through the comparator transistor. The comparator output node is initially set to *V*_DD_ and then gradually lowered due to the leakage. This output voltage drop limits the exposure time because the frame should be completed before the logic state of the comparator output changes due to leakage. Moreover, the leakage current entering the PD opposes the photocurrent, and it becomes worse as the PD voltage decreases during the exposure, degrading the low-illumination performance. These two issues prevent the use of larger *T*_S_ and *T*_F_ for the low-light-intensity range.

**Figure 6:**
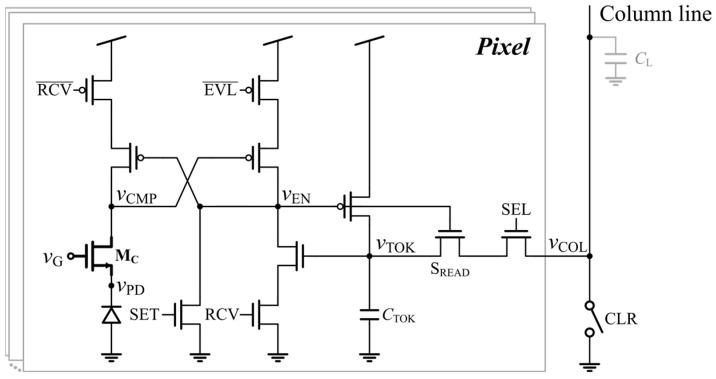
Architecture and schematic diagram of the proposed time-mode digital pixel CIS.

Figure 6 shows the schematic diagram of the proposed digital pixel CIS. The PD voltage *v*_PD_ is converted to a digital level by the comparator transistor M_C_, while all subsequent transistors operate as dynamic-logic switches. M_C_ is sized to a W/L of 0.48/0.18 μm to ensure sufficient comparator gain. A few mega-ohms of switch on-resistance is sufficient for nodal capacitances of a few femtofarads to ensure reliable dynamic-logic operation at a 20 MHz clock frequency. All switches use the minimum size with a W/L of 0.22/0.18 μm, and the on-resistances are simulated to be 15.6 kΩ (NMOS) and 48.6 kΩ (PMOS) at a 1.2 V supply voltage. As the resistances are far below the required range, the switch nonidealities have negligible influence on logic signals.

As all the signals other than the comparator-associated nodes operate at full logic levels, the charge injection from the switches does not affect the logic-state integrity. Moreover, the logic levels are restored during each recovery (RCV) phase incorporated in the proposed pixel, ensuring robust dynamic-logic operation. Among these switches, the READ switch is turned off only once at the beginning of the reset phase in each frame, and the charge injected into the token memory capacitor *C*_TKN_ is removed by the PMOS switch, restoring the logic-high level before the reset phase completes.

**Figure 7:**
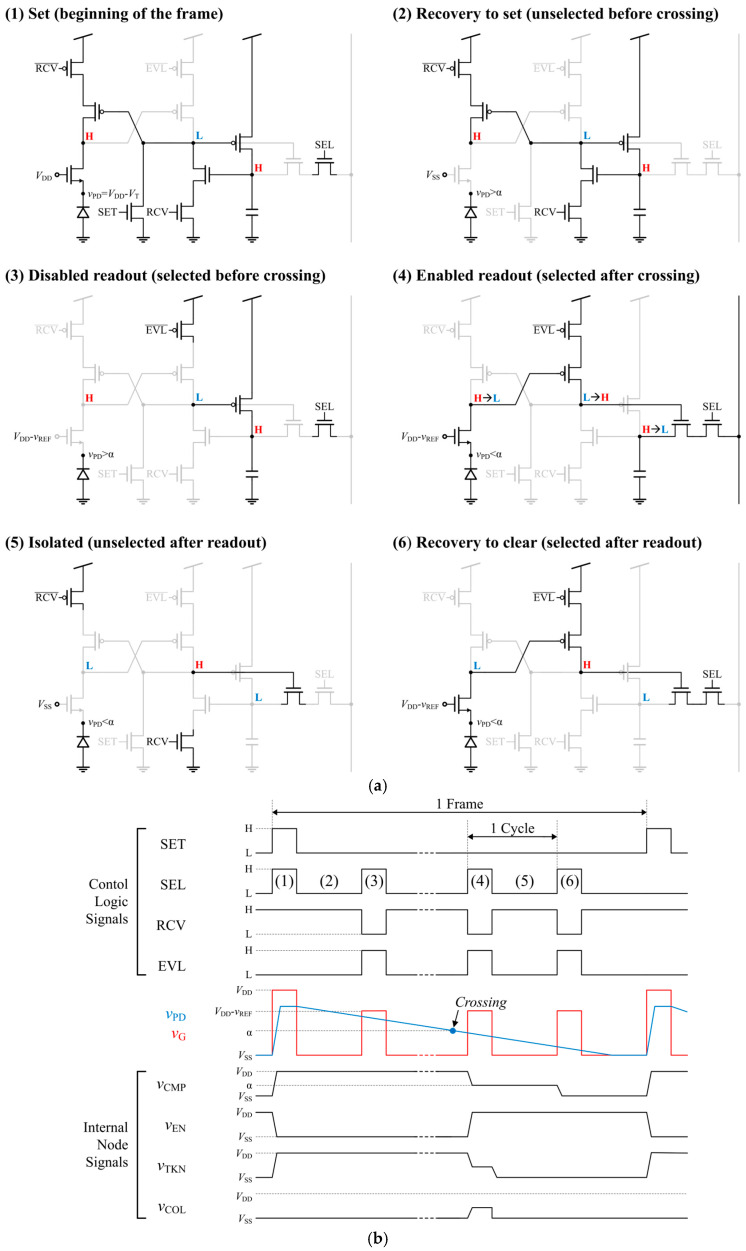
Operation of the proposed time-mode digital pixel CIS with α representing *V*_DD_-*v*_REF_-*V*_T_: (**a**) six operation phases and (**b**) timing diagram.

The digital pixel undergoes six distinct states, as shown in Figure 7. (1) ***Set***: At the beginning of each frame, the comparator output voltage *v*_CMP_ and the token capacitor voltage *v*_TOK_ are set to a high level. The PD voltage *v*_PD_ is set to *V*_DD_-*V*_T_ by applying *V*_DD_ to the gate voltage *v*_G_ of the M_C_. (2) ***Recovery to set***: When the pixel is unselected, only the RCV switches remain active, maintaining *v*_CMP_ and *v*_TOK_ at a high level. Meanwhile, the comparator is disabled by grounding *v*_G_, thereby blocking the leakage path. (3) ***Disabled readout***: When the pixel is selected, it operates in the evaluation phase. The EVL and select (SEL) switches are enabled, and *v*_G_ is restored to *V*_DD_-*v*_REF_, where *v*_REF_ is the reference voltage that changes as illustrated in Figure 3. The comparator remains off before the crossing, and the dynamic logic states are preserved because the READ switch blocks the token from being accessed. (4) ***Enabled readout***: Once *v*_PD_ in the selected pixel has crossed *V*_DD_-*v*_REF_*-V*_T_, the M_C_ turns on, pulling *v*_CMP_ down to a low level. Then, *v*_EN_ is pulled up to a high level, turning on the READ switch. At this moment, the token capacitor discharges its stored charge onto the column line, enabling token readout. (5) ***Isolated***: The pixel is isolated after token readout, even though the READ and RCV switches are on, because the token has been cleared. (6) ***Recovery to clear***: Any small change in *v*_TOK_ caused by leakage or charge injection after readout is cleared again whenever the pixel is selected by spilling the charge into the column line.

**Figure 8:**
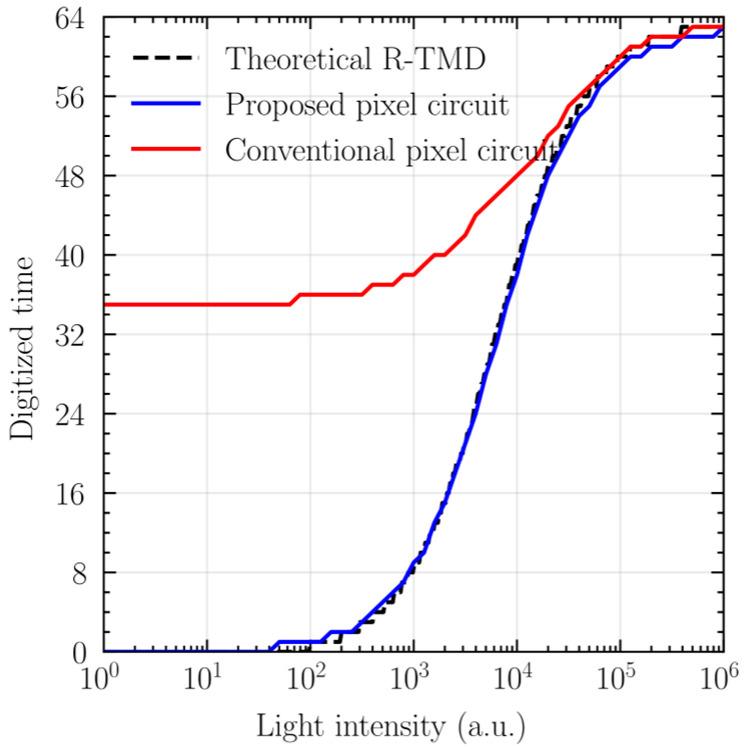
Transistor-level simulated photo-transfer curve comparison.

Figure 8 shows the photo-transfer curve obtained from the transistor-level simulations of the conventional pixel circuit [11] and the proposed low-leakage pixel circuit for 6-bit digitization. At low illumination, the conventional pixel circuit fails to utilize the full range of digital values due to the leakage current, which causes premature latching. In contrast, the proposed pixel circuit represents the full range of digital values, as expected.

Despite the CDS operation described above, non-idealities associated with the M_C_ cause fixed-pattern noise (FPN) and random noise (RN). First, *v*_PD_ cannot be completely charged to *V*_DD_-*V*_T_ during the *set* phase due to the finite duration. Second, the charge injection from the comparator transistor disturbs the *v*_PD_ when the M_C_ is turned off after the set phase. Third, the leakage current flowing through the comparator transistor is accumulated in the PD during the EVL phase. An incomplete CDS operation due to these three uncertainties causes worse FPN performance than that of a 4T-APS pixel or FPN-suppressed 3T-APS [13]. Since all other transistors, except the M_C_, operate as logic switches, their noise contribution is negligible. However, the intrinsic noise of the PD and the M_C_ is a major contributor to the RN in the image. In particular, flicker noise leads to larger random noise in the low-light-intensity region because it takes longer until the crossing occurs.

## 4. Measurement Results

**Figure 9:**
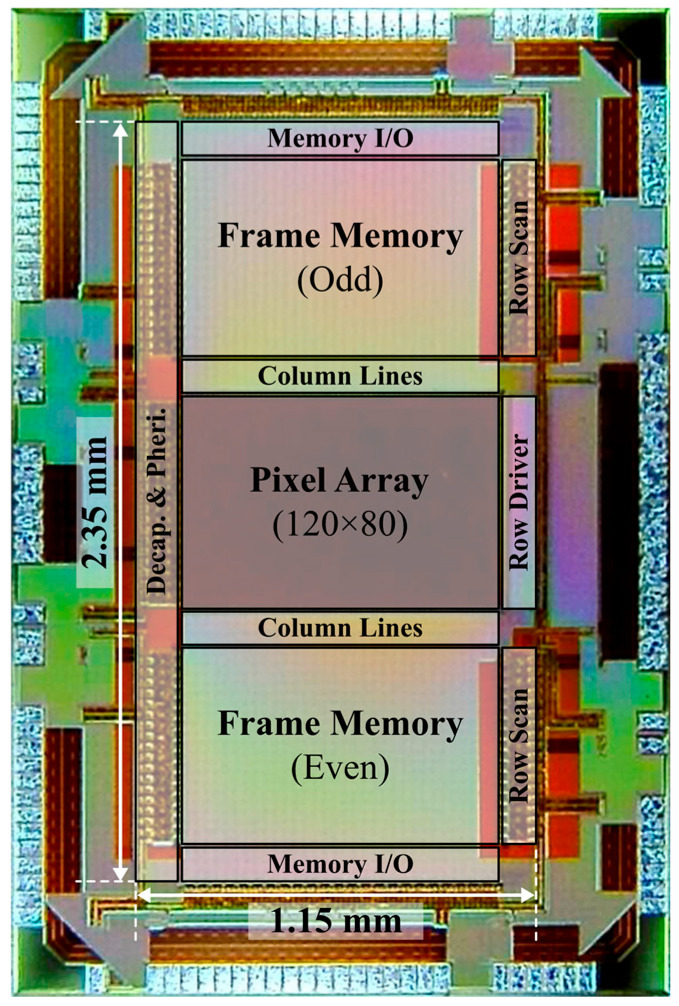
Chip micrograph.

The prototype chip was fabricated in a 0.18 μm standard CMOS process, as shown in Figure 9. The prototype includes the proposed pixel circuit array and a dual-port frame memory. The designed pixel pitch is 8 μm with a 17.5 μm^2^ n-well/p-sub PD, which is limited by the memory size rather than the pixel circuit. The pixel pitch and fill factor can be improved by adopting a 3D stacking technology [12] or embedded memory technologies, such as RRAM or 1T-SRAM [14,15]. The gate voltage for the comparator transistor was generated using an off-chip DAC controlled by the FPGA in this test chip, though it can be implemented as an on-chip multi-slope ramp generator.

**Figure 10:**
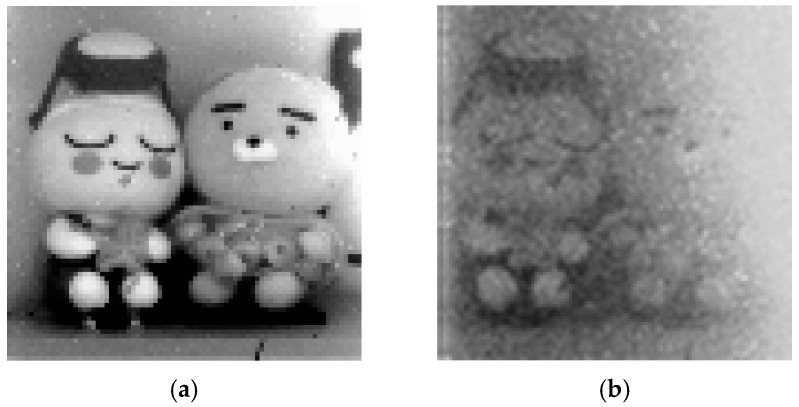
Captured sample images: (**a**) 190 lx, *T*_S_ = 17 ms, *t*_CK_ = 50 µs; (**b**) 1.9 lx, *T*_S_ = 400 ms, *t*_CK_ = 2 µs.

Figure 10 presents sample images captured under parameter settings optimized to represent the full illumination range of the scene. The sensor is configured to initiate counting after *T*_S_ for 2^6^ clock cycles while ramping down the reference voltage, thereby mapping the illumination range from 1/*T*_S_ to 1/[2^6^(*T*_S_ + 2^6^*t*_CK_)] into 6-bit digital data. The exposure time of Figure 10a corresponds to 1/50 s, whereas the exposure time of Figure 10b corresponds to 1/2.5 s to capture images at two orders of magnitude lower illumination. These results verify that the proposed pixel circuit effectively suppresses leakage, thereby allowing for operation with a large *T*_S_. However, the use of a large *T*_S_ increases the flicker-noise component, as shown in Figure 10b.

**Figure 11:**
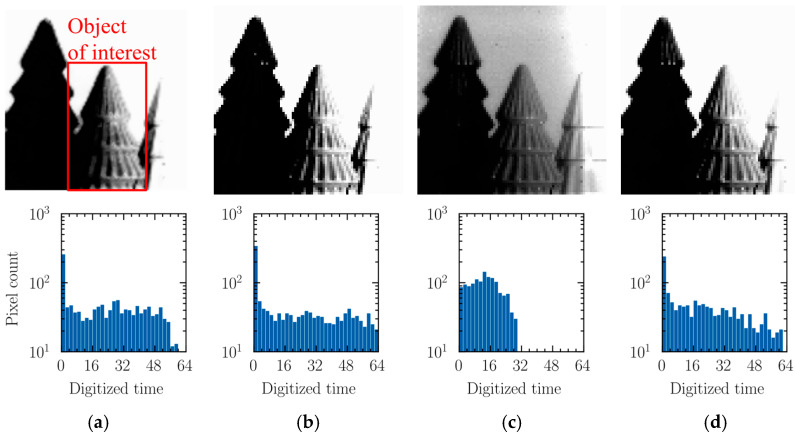
Captured sample images and their histograms: (**a**) 20.5 lx, *T*_S_ = 32.6 ms; (**b**) 200.6 lux, *T*_S_ = 4.6 ms; (**c**) 2106 lux, *T*_S_ = 0.64 ms; (**d**) 2106 lux, *T*_S_ = 0.64 ms, *T*_U_ = 0.74 ms, *T*_D_ = 1.84 ms, Δ = 0.5.

Figure 11 presents sample images captured with parameter settings optimized to emphasize the contrast for the object of interest in the middle of the image. The images were obtained under progressively increasing illumination levels, and the corresponding histograms for the object of interest are presented along with each image. Figure 11a,b demonstrate that the contrast of the intended object can be preserved by a properly selected *T*_S_ for the relatively low-illumination range. As the illumination increases, the histogram becomes compressed to a half scale due to limited time resolution, as shown in Figure 11c. The contrast for the intended object can be enhanced by introducing the ramp-up and flat phases, thereby extending the histogram to nearly full scale, as shown in Figure 11d.

Table 1 summarizes the performance of the proposed E-TMD CIS along with other state-of-the-art CISs (one low-power VMD CIS and three TMD CISs). Even though the FPN and the RN are higher than those of the other CISs, both remain below 1 LSB, which is sufficient for IoT applications. A major advantage of TMD CISs is their extended DR and low-power consumption. The DR figure of merit (FoM) is defined as a normalized DR with respect to the DR of an ideal N-bit digital value. Therefore, the DR FoM of a linear VMD CIS is close to 1, whereas that of a TMD CIS approaches 2, reflecting its extended DR. The DR FoM of the proposed E-TMD ranges between 1 and 2, depending on the choice of *T*_S_ and *T*_F_, and it was measured to be 1.6, with a *T*_S_ = 0.64 ms and *T*_F_ = 10.72 ms for 61 dB DR. Note that the TMD CISs exhibited a better power FoM than the state-of-the-art low-power VMD CIS. Although [11] reports a significantly improved power FoM, it is applicable for relatively high illumination above 0.7 lx. The proposed E-TMD achieves a 24-times improvement in minimum detectable light intensity down to 0.03 lx. The low-illumination performance is comparable to other CISs while maintaining a power FoM similar to that of state-of-the-art low-power TMD CISs.

## 5. Conclusions

This study presented an extended time-mode digital pixel CIS tailored for IoT applications. By introducing the E-TMD technique, the photo-transfer curve can be readily fitted to accommodate a wide range of illumination while providing sufficient contrast with a small digital bit depth. The proposed low-leakage digital pixel circuit enables TMD operation under a low-illumination region. The experimental results demonstrated that an E-TMD digital pixel sensor with a small digital bit depth can be used in various IoT image sensor systems.

## Figures and Tables

**Table 1:** Performance comparison of the proposed and state-of-the-art CISs.

Parameter	Park [7] JSSC’20	Chiou [10] JSSC’19	Kim [11] ISSCC’22	Rüedi [12] SSCL’24	This Work
Feature size [nm]	110	180	250	65	180
Process	CIS	CMOS	CMOS	CIS/3D stack	CMOS
Pixel supply [V]	2.5	0.4	1.2	1.8	1.2
Pixel array	640 × 640	300 × 200	120 × 120	640 × 480	120 × 80
Pixel pitch [µm]	4	7.6	8	6.3	8
Bit depth	10	12	8	10	6
Digitization mode	VMD	R-TMD	R-TMD	R-TMD	E-TMD
Frame rate [fps]	44	40	40	30	120
FPN [%_rms_]	N/A	0.16	0.64	0.60	1.62
RN [%_rms_]	0.05	0.10	0.19	N/A	0.24
DR [dB]	67	141	92	120	61
DR FoM *	1.1	1.9	1.8	1.9	1.6
Power FoM **	117	67	23	144	37 ***
Minimum intensity [lx]	N/A	0.005 ****	0.7	N/A	0.03

* DR FoM [-]: (DR)/(6.02 × Bit depth + 1.76). ** Power FoM [pJ/frame·pixel]: (Power excluding I/O)/(Total pixel number × Frame rate). *** Includes dual-port frame memory. **** Estimated from the transfer curve.

## Data Availability

The data presented in this study are available upon request.

## Abbreviations

The following abbreviations are used in this manuscript:

CMOS	Complementary Metal-Oxide Semiconductor
IoT	Internet of Things
CIS	CMOS Image Sensor
4T-APS	Four-Transistor Active Pixel Sensor
BSI	Back-Side Illumination
DR	Dynamic Range
PD	Photodiode
FD	Floating Diffusion
ADC	Analog-to-Digital Converter
TMD	Time-Mode Digitization
VMD	Voltage-Mode Digitization
FPN	Fixed-Pattern Noise
RN	Random Noise
RRAM	Resistive Random-Access Memory
1T-SRAM	One-Transistor Static Random-Access Memory
FoM	Figure of Merit
I/O	Input/Output
